# The remodeling of ovarian function: targeted delivery strategies for mesenchymal stem cells and their derived extracellular vesicles

**DOI:** 10.1186/s13287-024-03704-5

**Published:** 2024-03-27

**Authors:** Yinhua Song, Jiachen Wu, Yang Liu, Na Xu, Hualin Bai, Lingjuan Wang, Jihui Ai, Kezhen Li

**Affiliations:** 1 Department of Gynecological Oncology, Tongji Hospital, Tongji Medical College, Huazhong University of Science and Technology, Wuhan, 430030 Hubei China; 2 National Clinical Research Center for Obstetrics and Gynecology, Cancer Biology Research Center (Key Laboratory of the Ministry of Education), Tongji Hospital, Tongji Medical College, Huazhong University of Science and Technology, Wuhan, 430030 Hubei China; 3 Reproductive Medicine Center, Tongji Hospital, Tongji Medical College, Huazhong University of Science and Technology, Wuhan, 430030 Hubei China

**Keywords:** Mesenchymal stem cells, Extracellular vesicles, Premature ovarian insufficiency, Targeted delivery

## Abstract

Premature ovarian insufficiency (POI) is an essential cause of reduced fertility and quality of life in young women. Mesenchymal stem cells (MSCs) and MSCs-derived extracellular vesicles (EVs) have the ability to migrate to damaged tissues and are considered as promising therapeutic approaches for POI. However, the homing ability and therapeutic efficacy of MSCs administered in vivo are still insufficient, and their potential tumorigenicity and multi-differentiation potential also bring many doubts about their safety. The targeting ability and migration efficiency of MSCs can be improved by genetic engineering and surface modification, thereby maximizing their therapeutic efficacy. However, the use of viral vectors also has increased safety concerns. In addition, EVs, which seem to be the current therapeutic alternative to MSCs, are still poorly targeted for distribution, although they have improved in terms of safety. This paper reviews the comparative therapeutic effects of MSCs and their derived EVs on POI, their biodistribution after in vivo administration, and the most important possible ovarian targeting strategies. Difficulties such as homogeneity and yield before clinical application are also discussed. This article will provide new insights into precision therapy and targeted drug delivery for female ovarian diseases.

**Graphical Abstract:**

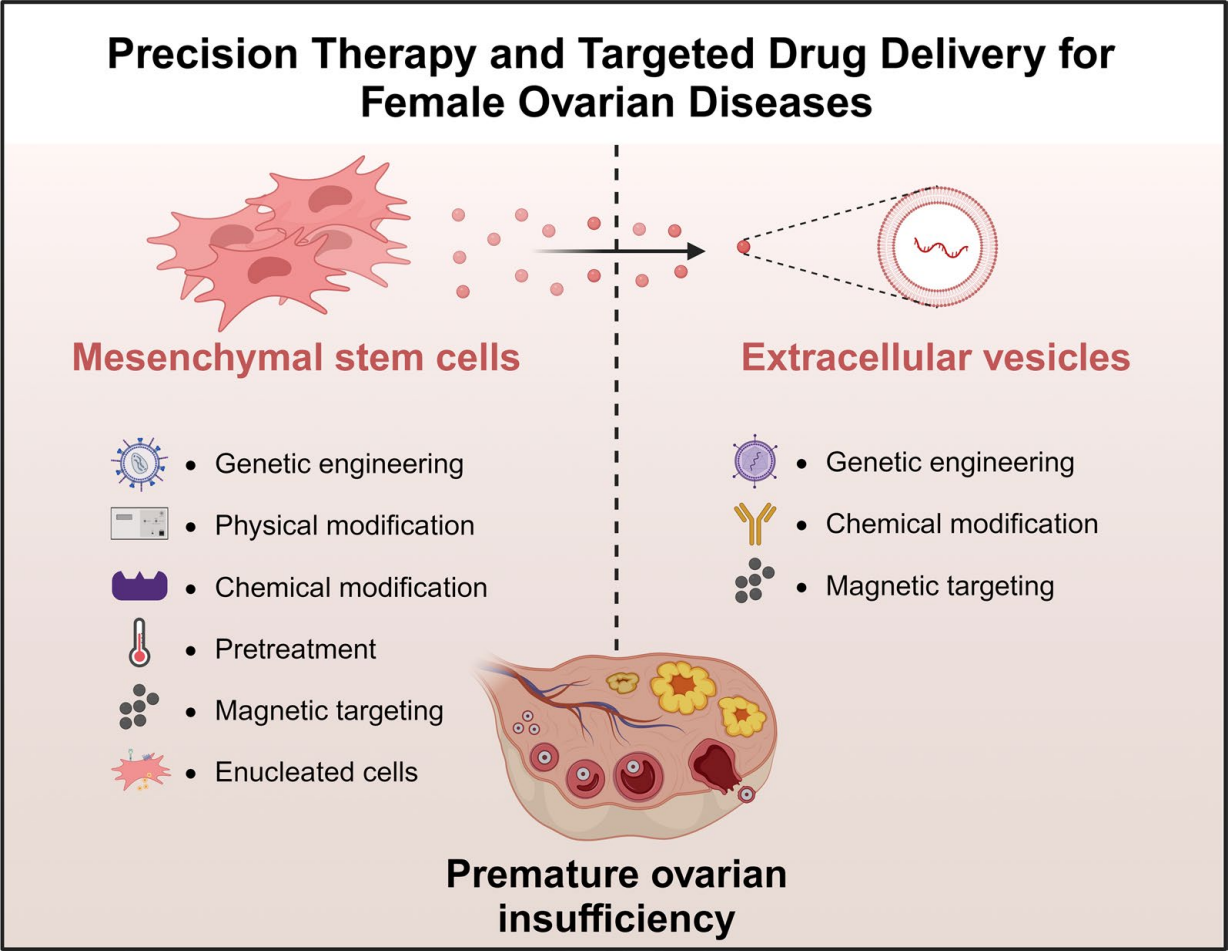

## Introduction

Normal ovaries in young women are essential for maintaining normal endocrine function and reproductive function in women, and their premature decline in function will reduce a woman's quality of life and lead to decreased fertility, with further progression leading to premature ovarian insufficiency (POI)[1]. Premature ovarian insufficiency (POI) is defined as a clinical syndrome of ovarian hypofunction in women before the age of 40 years, typically characterized by sporadic menstruation/amenorrhea, sex hormone deficiency, elevated serum gonadotropin levels, decreased libido, and loss of fertility, with further progression leading to premature ovarian failure (POF) [2]. It is reported that the prevalence of POI in women under the age of 40 is approximately 1% [3]. The occurrence of POI is associated with many complex factors, and the known causes include (1) genetic abnormalities, (2) autoimmune diseases, (3) surgical, radiotherapy or chemotherapy treatments, and (4) environmental toxins [4]. In the current social context, the high prevalence of POI may exacerbate the process of population aging and raise social concerns.

At present, preventive and therapeutic measures for POI are extremely limited, and the main clinical intervention is hormone replacement therapy (HRP) as a treatment to alleviate the symptoms of estrogen deficiency. However, HRP is not effective in restoring ovarian function and even has the risk of inducing estrogen-dependent tumors [5, 6]. Although assisted reproductive technology (ART) such as in vitro fertilization-embryo transfer (IVF-ET) is very well established, it is not suitable for the treatment of POI because it requires women to provide high-quality oocytes [7]. For POI patients who desperately want to have offspring, in vitro activation (IVA) can be performed by surgically removing a portion of the ovarian cortex, activating the primordial follicle in vitro, and transplanting it back into the body to continue development and ovulation [8]. However, the disadvantages of this technique are the invasiveness of the surgery, the adverse effects of tissue culture on the follicles, and the need for further studies to confirm its efficacy and safety [9]. Therefore, there is an urgent need to explore safe and effective therapies for remodeling of ovarian function.

With the application of mesenchymal stem cells (MSCs) and their related technologies in regenerative medicine, MSCs have shown promising therapeutic effects in neurological disorders, pulmonary dysfunction, metabolic/endocrine-related diseases, skin burns, and cardiovascular diseases, and they are gradually becoming the most promising therapeutic option in ovarian function reconstruction research [10–12]. The prevailing view is that the mechanism of MSCs for the treatment of POI is mainly based on their paracrine effects after migration to the ovary, including the secretion of cytokines, chemokines, growth factors, and extracellular vesicles (EVs), which play a regulatory role in the proliferation, apoptosis, immunity, autophagy, oxidative stress, angiogenesis and fibrosis of ovarian cells [13–15].

EVs are membrane vesicles 50–1000 nm in diameter that are secreted by almost all cell types and contain proteins, lipids, and genetic material [16]. Based on the known mode of biogenesis, EVs can be categorized into three main types: exosomes, microvesicles and apoptotic bodies [17]. Exosomes bud inward from endosomes to form intraluminal vesicles (ILVs) in multivesicular endosomes (MVEs), which are released by MVEs fusion with the plasma membrane (30–120 nm in diameter). Microvesicles are formed by budding outward directly from the plasma membrane (150–1000 nm in diameter). Apoptotic bodies are generated during programmed cell death (100–5000 nm in diameter).

EVs are thought to be a mechanism for intercellular communication, allowing cells to exchange substances and participate in a variety of physiological and pathological processes [18]. Notably, EVs can be used as a novel transport vehicle to transport microRNAs (miRNAs), small interfering RNA (siRNAs), or chemotherapeutic drugs to target organs, and are now a new generation of diagnostic and therapeutic tools in the field of nanomedicines.

MSCs and MSCs-derived EVs have gradually become the most promising option for POI treatment [19]. However, in addition to the inherent safety limitations of MSCs themselves, the shortcomings of many of the current POI-related studies are the failure to address the non-targeted distribution of MSCs and MSCs-derived EVs in various organs with minimal distribution in the ovary after intravenous injection, especially the blockage in the lungs of MSCs, and the failure to capitalize on the properties of EVs as a nanomedicine carrier [20–22]. Nowadays, the concept of precision medicine is deeply rooted, and the development of targeted therapies and precision drug delivery systems for ovaries by MSCs and MSCs-derived EVs is highly promising and challenging.

This review starts from the recent research advances regarding MSCs and MSCs-derived EVs for remodeling of ovarian function and describes the homing mechanisms of MSCs and MSCs-derived EVs. Importantly, this article provides insight into possible ovarian-targeted homing strategies as well as points to note.

## Comparison of the therapeutic effects of MSCs and MSCs-derived EVs on POI

According to the definition of the International Society for Cellular Therapy (ISCT), MSC is a pluripotent progenitor cell with the ability to renew itself (limited in vitro) and differentiate into mesenchymal cells [23]. MSCs-derived EVs carry substances such as nucleic acids, proteins, lipids, and metabolites from parental cells, and their therapeutic potential for POI has now been demonstrated. Relevant basic studies in recent years have shown that MSCs and their derived EVs have similar therapeutic effects on POI [24–45] (Tables 1, 2).

**Table 1:** Therapeutic effects and mechanisms of MSCs on POI in different models

Type of MSCs	Model	Route of administration	Outcome	Mechanism	References
hUMSCs	CDDP	Tail vein injection	Ovarian fibrosis↓	TGF-β1/Smad3	[24]
	CDDP	Tail vein injection	Theca-interstitial cells apoptosis ↓ Oxidative stress ↓	AMPK/mTOR pathway	[25]
	CDDP	Tail vein injection	Ovarian fibrosis ↓	AMPK/NR4A1 pathway	[26]
	CTX and BUS	Tail vein injection	Ovarian metabolome ↑	PI3K pathway	[27]
	CTX and BUS	Tail vein injection	GCs apoptosis ↓ Inflammation ↓	P38 and AKT pathway	[28]
hAMSCs	CTX	Tail vein injection	GCs apoptosis ↓ Angiogenesis ↑	Bax, Bcl2 and VEGF	[29]
	CTX	Tail vein injection	GCs apoptosis ↓ Angiogenesis ↑	SDF-1/CXCR4 axis; PI3K/Akt pathway	[30]
	10% hydrogen peroxide	Intraperitoneal injection	Fertility ↑ Inflammatory cytokines ↓	–	[31]
mBMSCs	CTX	Situ ovarian injection	GCs apoptosis ↓	Overexpression of miR-21; PDCD4 and PTEN	[32]
	ϒ-radiation	Tail vein injection	Ovarian apoptosis ↓ Ovarian proliferation ↑	TGF-β, Wnt/β-Catenin and Hippo pathway	[33]
MenSCs	CDDP	Tail vein injection	GCs apoptosis ↓ Ovarian fibrosis ↓	Secret FGF2	[34]

**Table 2:** Therapeutic effects and mechanisms of MSCs-derived EVs on POI in different models

EVs cellular origin	Method	Route of administration	Outcome	Mechanism	References
hUMSCs	CDDP	Tail vein injection	GCs apoptosis ↓ angiogenesis ↑	miR-126-3p via PI3K/AKT/mTOR pathway	[35]
	CTX	Tail vein injection	Angiogenesis ↑	PI3K/AKT pathway	[36]
	CDDP	Tail vein injection	GCs apoptosis ↓	MicroRNA-22-3p via KLF6 and ATF4-ATF3-CHOP pathway	[37]
	CTX	Tail vein injection	GCs proliferation ↑ ROS accumulation ↓	MicroRNA-17-5p via SIRT7 pathway	[38]
	Aging mouse	Tail vein injection	Oocytes activation ↑ Fertility ↑	miR-146a-5p and miR-21-5p via PI3K/mTOR pathway	[39]
	CDDP	Tail vein injection	GCs apoptosis ↓	MicroRNA-29a via HMG-Box /Wnt/β-Catenin pathway	[40]
hADSCs	CTX	Tail vein injection	GCs proliferation ↑ GCs apoptosis ↓ GCs marker ↑	SMAD pathway	[41]
mBMSCs	CDDP	Tail vein injection	GCs apoptosis ↓	miR-644-5p via P53 pathway	[42]
hAMSCs	CTX	Tail vein injection	GCs proliferation ↑ GCs apoptosis ↓ ROS accumulation ↓	SIRT4 pathway	[43]
hAFSCs	CTX	Tail vein injection	GCs apoptosis ↓	miR-369-3p via YAF2/PDCD5/p53 pathway	[44]
iPSC-MSC	CTX	Tail vein injection	GCs proliferation ↑ GCs apoptosis ↓	ILK-PI3K/AKT pathway	[45]

**Fig. 1:**
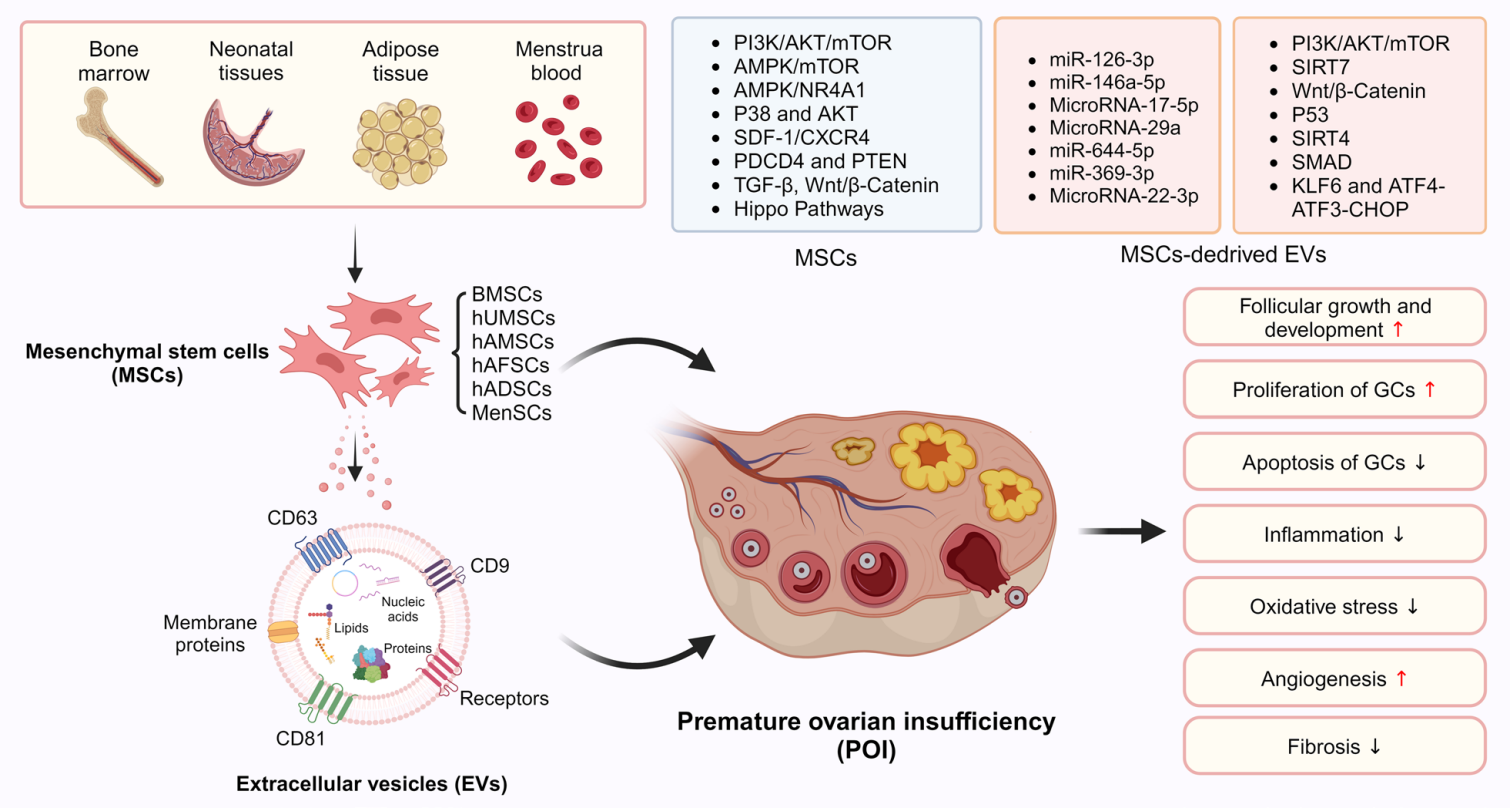
The mechanisms of MSCs and MSCs-derived EVs for POI treatment

The mechanisms of MSCs and MSCs-derived EVs for POI treatment can be summarized as follows (Fig. 1): (1) promoting follicular growth and development; (2) promoting proliferation of GCs or inhibiting apoptosis of GCs; (3) promoting ovarian angiogenesis; (4) immunomodulatory and anti-inflammatory effects; (5) reducing oxidative stress; and (6) inhibiting fibrosis.

The advantage of MSCs over EVs is their ability to survive, proliferate, and differentiate in vivo, which may have a longer-term and better therapeutic effect. However, as research progresses, the drawbacks of MSCs transplantation in vivo have been revealed, such as safety of preparation, unpredictable differentiation after in vivo transplantation, potential tumorigenicity, donor or tissue source heterogeneity, ethics, and stem cell regulation [46, 47]. In contrast, EVs have higher biological stability, lower immunogenicity, no risk of live cell administration, easier to produce on a large scale at low cost and without potential ethical issues [48].

In addition to the above advantages and disadvantages, a common disadvantage of both is the poor homing effect of target organs after systemic injection. While the potential effect of non-target organ homing is currently unknown, improving the homing ability of MSCs and their EVs is of greater basic research value and clinical application potential.

## Biodistribution of MSCs and MSCs-derived EVs

Routes of administration of MSCs and EVs for POI routinely include intravenous and in situ, with a few using arterial injections. Among them, intravenous injection is the simplest, safest, and low-cost method, and therefore the most commonly used and more suitable for clinical application. Unfortunately, the homing efficiency of MSCs or EVs after intravenous injection is extremely low and only a very small fraction of them will reach the ovaries. This is a major bottleneck for MSCs or EVs in the treatment of POI and for future clinical applications.

### Biodistribution of MSCs

**Fig. 2:**
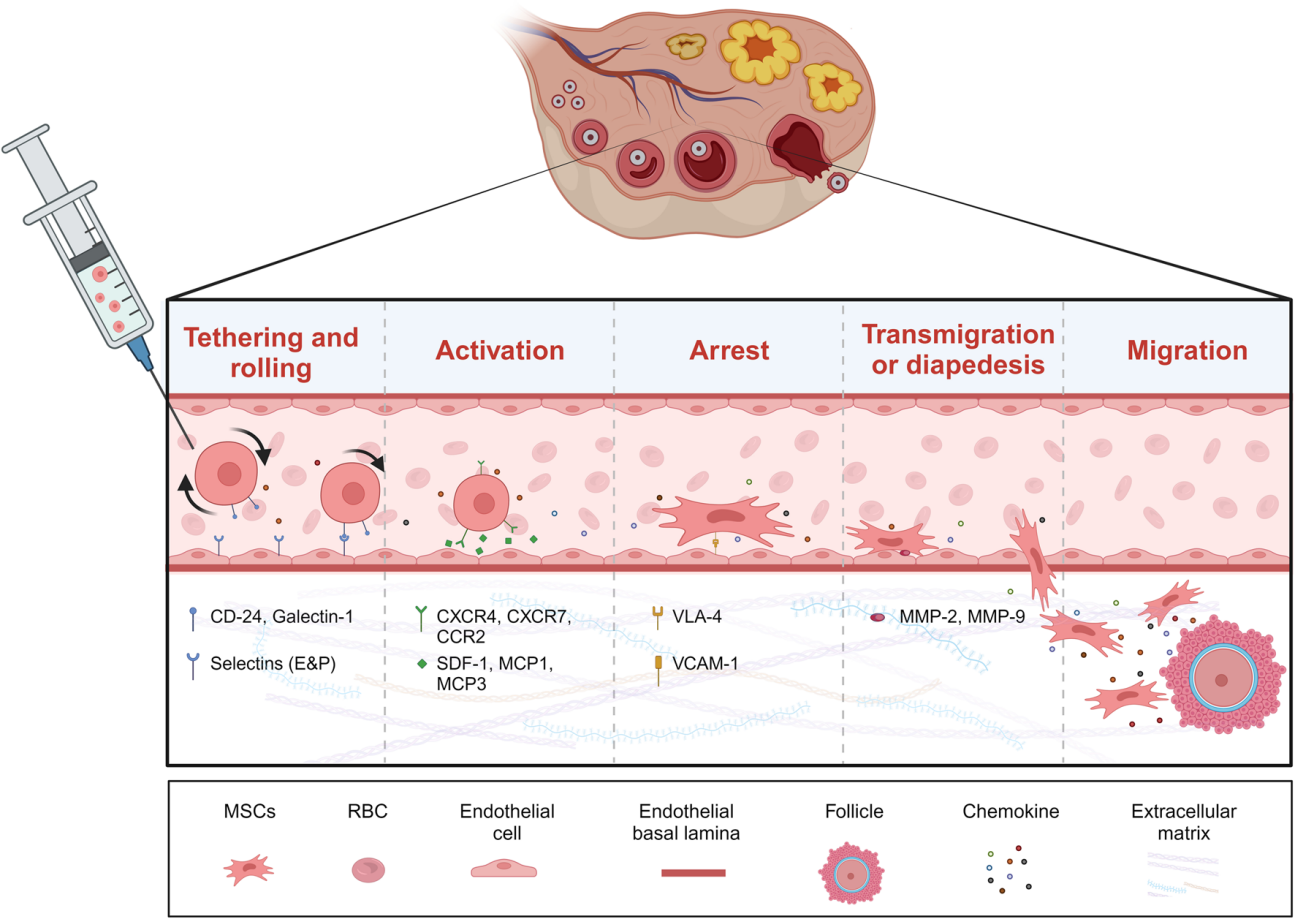
The mechanisms of MSCs homing

Although the current study suggests that MSCs leave the circulation through a leukocyte homing-like mechanism mediated by specific receptor-ligands and undergo five steps of (1) tethering and rolling, (2) activation, (3) arrest, (4) transmigration or diapedesis, and (5) migration to further migrate to the site of injury [49] (Fig. 2). This is the result of "active homing", but what is often not noted in many POI-related studies is the "passive homing" effect of MSCs. It has been shown that the cell volume of MSCs increases significantly in the context of in vitro wall culture. Unlike endogenous MSCs that circulate efficiently throughout the body, this exogenous large volume of MSCs is passively entrapped in large numbers in small-diameter vessels [22]. Studies have shown that approximately 99% of MSCs are clear from circulation within 5 min of intravenous injection, with over 80% of the cells being entrapped in the lungs and only extremely small amounts of MSCs in other organs [50]. This entrapment leads to the formation of local microemboli, causing local ischemia and resulting in massive death of MSCs, with only a few MSCs surviving in the perivascular niches [51]. Despite the mechanism of deformation, MSCs are not able to change the above fate due to physical limitations.

Arterial injection seems to be a good option to avoid the "first-pass effect" [52] in the lungs and to allow more MSCs to accumulate in the target organ. However, similar to the above mechanism, MSCs will inevitably form microemboli in local small vessels, resulting in the ischemic death of MSCs and the risk of local ischemic damage to the organ [51]. In addition, invasive surgery, bleeding complication, and concerns about the safety of irreversible ischemic damage to the ovaries will limit the clinical use of arterial injections.

For in situ injection, the ovary is a parenchymal organ, not a cavernous structure similar to a joint cavity [53], and it is not suitable to inject large amounts of MSCs, and forced injection would inevitably cause local mechanical damage to the ovary. In addition, many animal experiments have been performed based on the cystic envelope wrapped around the surface of the ovary in mice or rats, into which MSCs can be injected and survive for a short period, waiting for MSCs to migrate into the ovary or act on ovarian cells through paracrine mechanisms [32]. However, the human ovary is not encapsulated by such a cystic structure and intracapsular injection is not feasible. Therefore, the safety and feasibility of in situ injection needs to be thoroughly evaluated before its clinical application.

### Biodistribution of EVs

As an approach to cell-free drug delivery therapy, EVs have the characteristics of small size, low immunogenicity, long circulating half-life, good permeability, and good biocompatibility [54], which are very promising to be applied in the treatment of POI. Unfortunately, as with most other nanoparticle-based drug delivery vehicles, by only using the inherent properties of MSC-derived EVs, the precise treatment of POI and the targeted delivery of drugs could not be achieved.

**Fig. 3:**
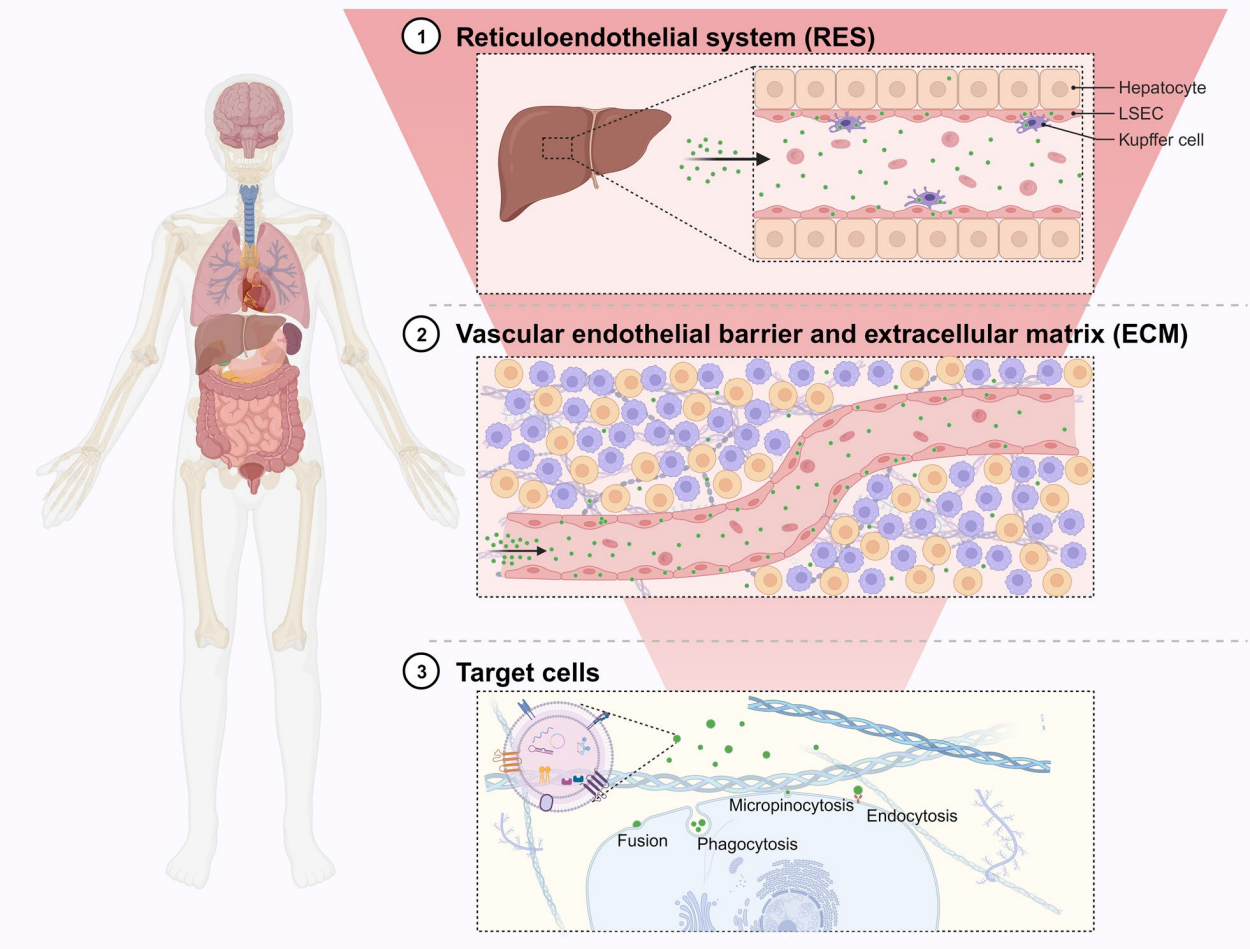
The mechanisms of EVs homing

**Fig. 4:**
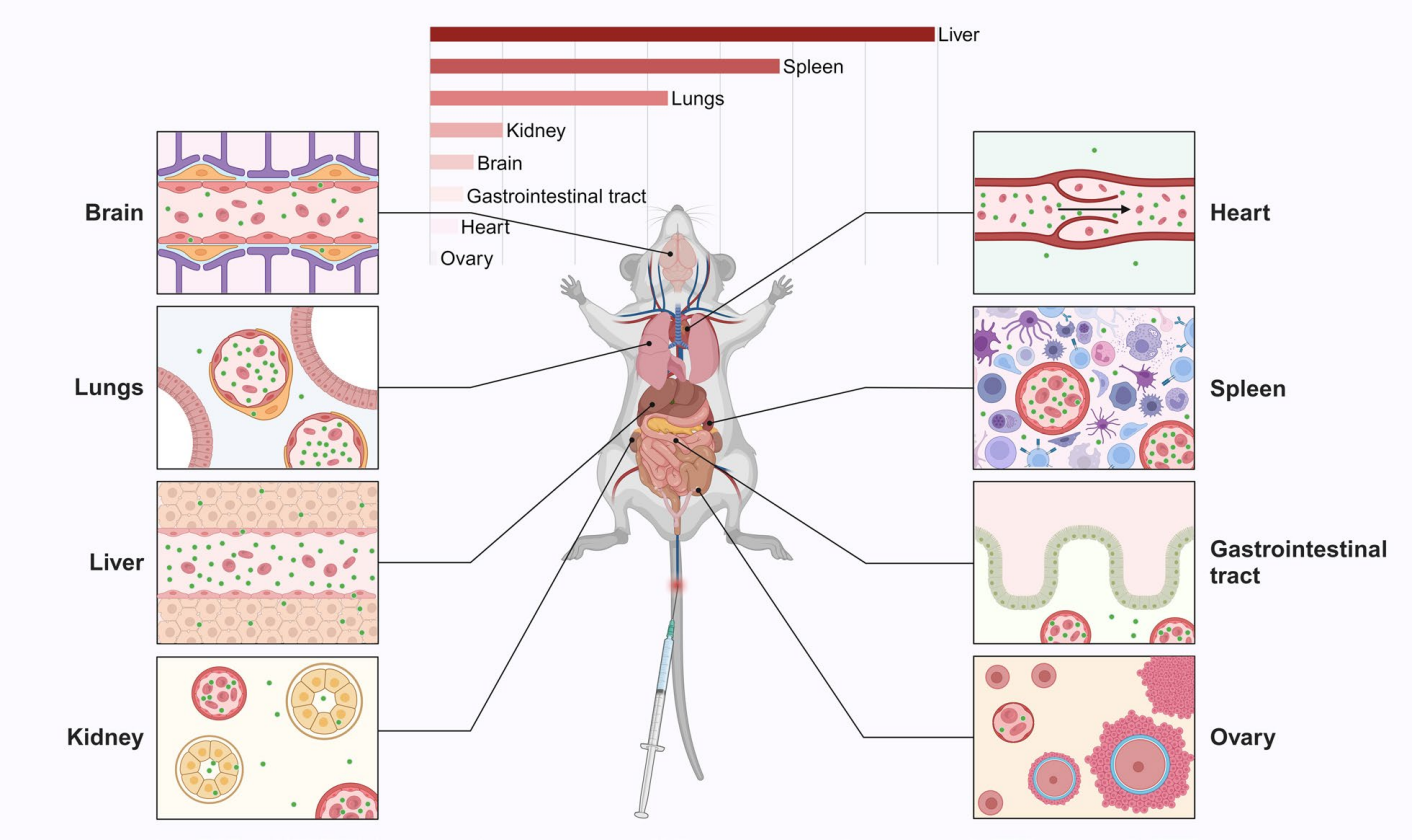
The distribution of EVs after intravenous injection

After intravenous injection, EVs typically undergo the following process, with only a small percentage eventually reaching the intended targets [55] (Fig. 3): (1) Flow in the circulatory system while being mostly removed to the liver and spleen of the reticuloendothelial system (RES); (2) cross the vascular endothelial barrier and extracellular matrix (ECM); (3) uptake by target cell (including phagocytosis, micropinocytosis, endocytosis and fusion) [56]. As with the above principles, many studies have shown that after intravenous injection, EVs accumulate most in the liver, spleen, lungs, and kidneys, with extremely low distribution in the ovaries [21, 57–59] (Fig. 4).

## Ovarian targeting strategies of MSCs

**Fig. 5:**
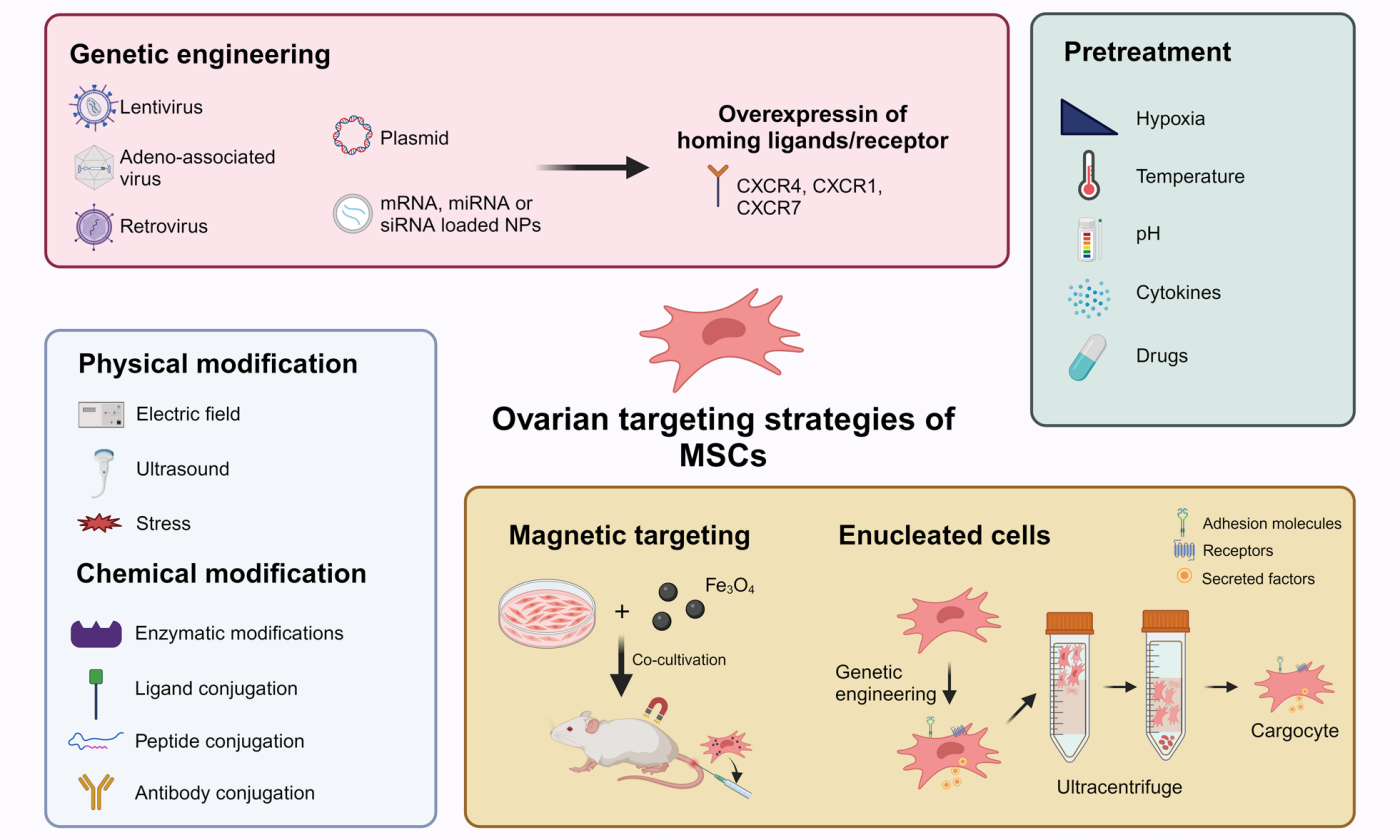
Ovarian targeting strategies of MSCs

In view of the above deficiencies in "active homing" and difficulties in "passive homing" of MSCs after injection, the main challenge is to improve the ovarian biodistribution of MSCs and to enhance the therapeutic effect. Here we discuss possible ovarian targeting strategies of MSCs (Fig. 5).

### Genetic engineering

Genetic engineering is a convenient tool to modify genes to improve the therapeutic potential of MSCs, including homing ability, anti-inflammatory ability, differentiation ability, angiogenesis, and survival time [60, 61]. In the "active homing" mechanism, the specific chemokine receptors on the surface of MSCs are key proteins for MSCs to recognize chemokines, and overexpression of chemokine receptors through genetic engineering can improve the homing ability of MSCs. For example, CXCR4 on the surface of MSCs has been shown to be involved in the migration and initiation of the homing process of MSCs through the chemokine SDF-1, and the expression of CXCR4 gradually decreases during in vitro culture [62]. It has been reported that ovarian and serum levels of SDF-1 are significantly elevated after chemotherapy, that ovarian induction of CXCR4-expressing MSCs homing, and that blocking the SDF-1/CXCR4 axis significantly reduces the number of MSCs homing [30]. In addition, overexpression of chemokine receptors, such as CXCR1 (IL-8), CXCR4 (SDF-1), and CXCR7 (SDF-1), has been shown to enhance the migration and targeting ability of MSCs [60].

Notably, the use of viral vectors, including retrovirus, lentivirus, and adeno-associated virus (AAV), inevitably carries the risk of chromosomal instability, insertional mutagenesis, and proto-oncogene activation, safety issues that hinder clinical use remain. To address the safety issues of viral vector genetic engineering, there have been attempts to integrate suicide genes in MSCs so that MSCs die after their therapeutic effects, avoiding virus-related risks [63–65]. In addition, non-viral transfection means can deliver DNA/RNA into cells by physical means or transfection reagents to achieve transient gene expression. Marquez-Curtis et al. used the cationic liposome reagent IBAfect to increase the expression of CXCR4 on the surface of MSCs and a significant 1.3-fold increase in the number of MSCs migrating toward the SDF-1 gradient [66]. Rosario et al. used transient transfection of mRNA to simultaneously overexpress CXCR4 as well as the anti-inflammatory cytokine IL-10, which enhanced the homing of MSCs to inflammatory sites and increased the anti-inflammatory effect [67]. However, in addition to mRNA transfection, safety concerns remain for non-viral transfected DNA.

### Physical modification

Physical modification refers to the stimulation of cells by physical stimulation, including electric field, ultrasound, and stress, which affects certain signaling pathways and thus regulates the expression of cell surface ligands/receptors. For example, low-intensity pulsed ultrasound (LIPUS) stimulation promotes the expression of hAD-MSCs chemokine receptors, especially CXCR4, and to some extent increases the number of hAD-MSCs homing to CTX-injured ovaries [68]. In other disease areas, the homing ability to cisplatin-induced damaged kidneys was enhanced in BMSCs exposed to electric field [69].

### Chemical modification

Chemical modifications including the use of enzymatic modifications and ligand/peptide/antibody conjugation could avoid the potential safety issues of genetic engineering and transiently improve the homing of MSCs, however, there are currently no studies in the field of POI. In a classic study, since hMSCs do not express E-selectin ligands, researchers used an enzyme preparation (a-1,3-fucosyltransferase preparation) to convert MSCs expressing CD44 into hematopoietic cell E- selectin/L-selectin ligand, thus giving MSCs the ability to bind E-selectin and ultimately allowing MSCs to migrate to the targets [70]. Conjugation of antibodies to MSCs is currently a popular strategy, for example, Sulaiman et al. used palmitated protein G (PPG) as a mediator and MSCs were successfully coupled to type II collagen antibody, increasing the ability to bind to the osteochondral surface [71]. Liao et al. increased the hepatic accumulation capacity and therapeutic efficacy of ADSCs by conjugating the targeting peptide RLTRKRGLK on the surface of ADSCs by a bioorthogonal click chemistry [72].

### Pretreatment

Preconditioning refers to briefly causing functional changes in MSCs with physiological conditions including hypoxia, temperature, and pH, or cytokines and small molecule drugs to enhance the repair effect or homing ability of MSCs. To address the problem of decreased survival of MSCs after in vivo injection, researchers used heat shock (HS) to pretreat MSCs and found that HS significantly reduced the apoptosis rate of MSCs and enhanced the repair effect on MSCs to chemotherapy-induced POI [73, 74]. Although hUMSCs pretreated with hypoxia can significantly reduce apoptosis in transplanted ovarian tissues and improve early pro-angiogenic effects [75], there are no relevant studies on whether they can improve ovarian homing efficiency. In other fields, upregulation of CXCR4 and CXCR7 expression in hypoxia-pretreated MSCs enhanced the homing ability and therapeutic effect in renal ischemia/reperfusion injury models [76]. BMSCs preincubated with tumor necrosis factor α (TNF-α) upregulated the expression of several chemokine receptors such as CCR2, CCR3, and CCR4, and increased migration toward chemokines [77].

### Magnetic targeting

Magnetic targeting refers to the use of magnetic fields to guide MSCs with magnetic nanoparticles to the organ of interest after intravenous injection to improve the distribution of MSCs in vivo. At present, the main target organs involved in MSCs magnetic targeting studies include spinal cord [78], lungs [79], spleen [80], brain [81], heart [82], knee joint [83] and retina [84]. For example, Liu et al. labeled MSCs with Fe_3_O_4_@polydopamine (PDA) and fixed magnets to the dorsal L4-L6 segment of the spinal cord of mice for 24 h, which improved the homing ability and therapeutic effect of MSCs to the spinal cord [78]. However, the placement of permanent magnets is a challenge considering the size of the ovary as well as the site, thus limiting the application of magnetic targeting to the ovary. In addition, some safety issues of magnetic targeting need to be addressed before conducting clinical trials, including biocompatibility issues between MSCs and magnetic nanoparticles, the effect of static magnetic fields on MSCs, and adverse effects in vivo [85].

### Enucleation

Wang et al. innovatively used Cytochalasin B and Ficoll density-gradient ultracentrifugation to remove the nuclei of hTERT-immortalized adipose-derived MSCs (hT-MSCs) and combined with in vitro 3D cell culture, which significantly reduces the cell diameter of MSCs and preserves key cellular structures and functions, such as translation of exogenous mRNAs and secretion of functional proteins. On this basis, the combination of genetic engineering techniques, such as overexpression of CXCR2, CXCR4, and PSGL-1/FUT-7 to enhance homing ability and overexpression of IL-10 to enhance anti-inflammatory ability, well addressed the homing challenges and unpredictability of differentiation of MSCs as well as the oncogenicity of genetic engineering [86]. The breakthrough effect of this technology is to solve the "first-pass effect" in lungs and cell safety problems and to preserve the key structure and function of cellular protein expression and secretion.

### Comparisons of ovarian targeting strategies of MSCs

Safety concerns are the first thing that needs to be considered for the remodeling of ovarian function in women. As previously mentioned, genetic engineering tools face safety concerns due to the use of viruses. In fact, physical modification, chemical modification, pretreatment, and magnetic targeting still involve the potential tumorigenicity of stem cells because they involve the in vivo injection of MSCs. In contrast, the expression of "suicide genes" by MSCs through genetic engineering can to a certain extent alleviate safety concerns, but the efficiency of cell suicide and the impact of viruses need to be considered. In addition, due to the specific location and size of the ovary, the difficulty of placing permanent magnets will greatly limit the application of magnetic targeting technology. Therefore, "decellularized" nucleated cells are relatively the best choice, on the one hand, it can be combined with the advantages of genetic engineering to achieve enhanced homing ability and anti-inflammatory ability, on the other hand, after the nucleation of the natural solution to the problem of cellular safety, and due to the smaller size of the cells, it can also reduce the “first-pass effect” in the lungs, greatly improving the targeting efficiency.

## Ovarian targeting strategies of MSCs-derived EVs

**Fig. 6:**
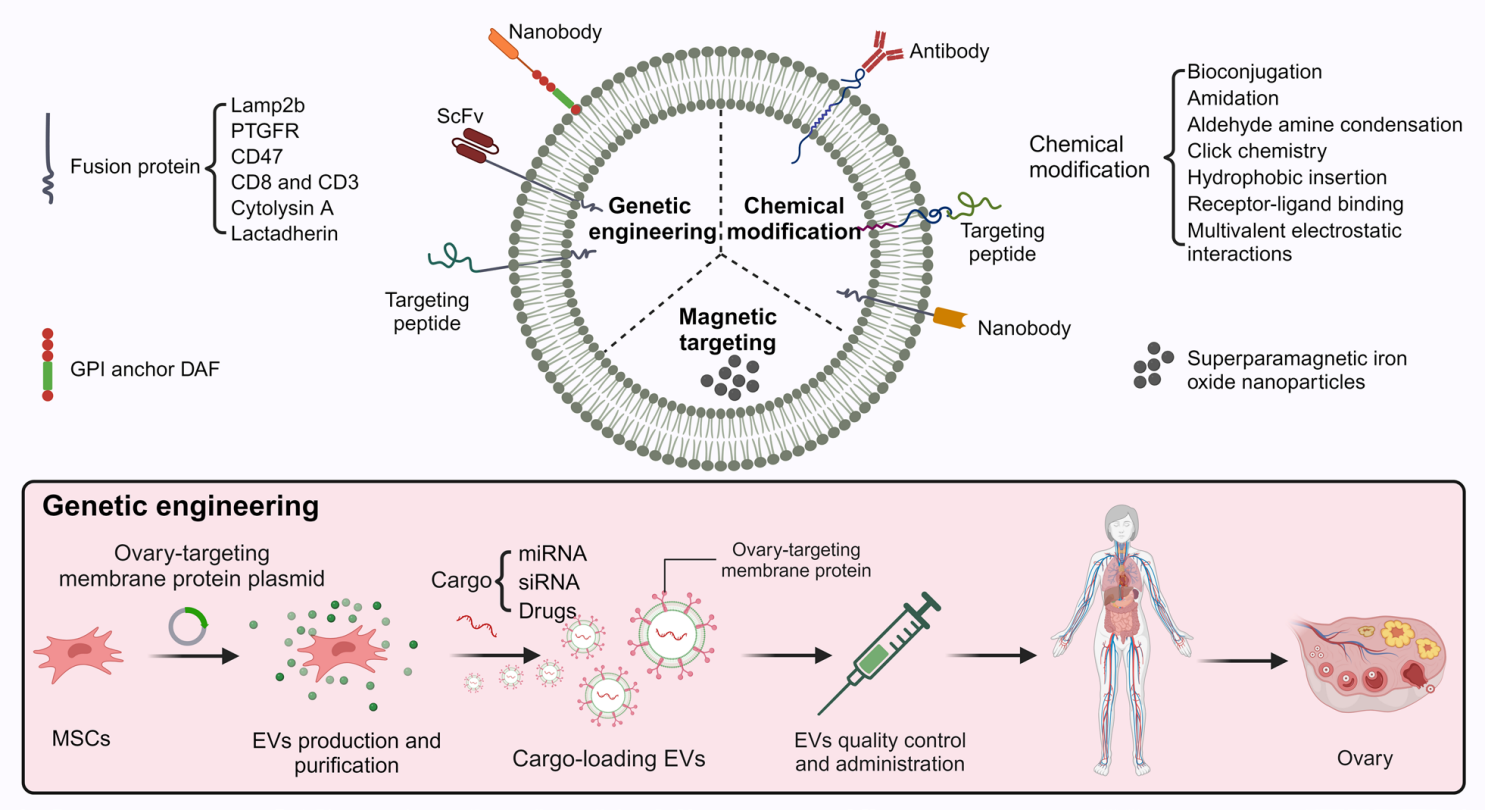
Ovarian targeting strategies of EVs

EVs, especially exosomes, are well suited as tools for ovarian-targeted therapies due to their favorable nano-properties. For example, EVs can cross the blood-follicle barrier to exert therapeutic effects [36]; there is a "do not eat me" signal CD47 and therefore a long circulating half-life [87]; MSCs derived EVs have inherent anti-inflammatory and repair properties [88]; and the contents of EVs, including nucleic acids, proteins, and even loaded drugs, can be modified to significantly increase the therapeutic potential of EVs [89]. Therefore, it would be of great interest to overcome the challenge of insufficient biodistribution and explore ovarian targeting strategies for EVs (Fig. 6).

At present, the main engineering strategies for EVs include cargo loading and membrane-targeted modification [90]. Cargo loading is achieved by co-incubation, genetic engineering, physical and chemical pathway-mediated membrane penetration, or membrane fusion. And the prerequisite to realize targeted cargo delivery lies in good membrane-targeted modification or magnetic targeting. The means of membrane-targeting modifications are mainly based on the membrane structure of the phospholipid bilayer of EVs. The proteins on the surface of EVs are manipulated through genetic engineering or chemical modification to express or conjugate single chain antibodies (scFv)/targeting peptide/nanobodies on the membrane surface of exosomes.

### Genetic engineering

The key after genetic modification lies in the fusion protein expressed on the surface of the exosome membrane, which has targeting ability. Alvarez-Erviti et al. [91] pioneered the introduction of a vector encoding a neuron-specific RVG peptide into cells for fusion expression with Lamp2b and successfully delivered exosomes loaded with siRNA to the brain for therapeutic effect. Similar examples of Lamp2b fusion-expressed targeting peptide include targeting of cardiomyocytes [92, 93], breast cancer cells [94], and chondrocytes [95]. Zou et al. [96] expressed human immunodeficiency virus type 1 (HIV-1) high-affinity specific single chain antibody fragment on the surface of exosomes and loaded curcumin or miR-143 to successfully target and kill virus-infected cells. Kooijmans et al. [97] introduced anti-epidermal growth factor receptor (EGFR) nanobodies with glycosylphosphatidylinositol (GPI) anchor signal peptides derived from decay-accelerating factor (DAF) fusion expression vectors into EVs-producing cells and successfully targeted tumor cells.

### Chemical modification

In contrast to genetic engineering, which requires parental cells, chemical modification is a direct intervention in EVs, including bioconjugation, amidation, aldehyde amine condensation, click chemistry, hydrophobic insertion, receptor-ligand binding, multivalent electrostatic interactions [90]. Cui et al. [98] through a diacyllipid insertion method conjugated the bone-targeting peptide SDSSD to MSCs derived exosomes membrane, and then the exosomes were loaded with siRNA via electroporation, successfully delivered siRNA specific targeting to osteoblasts, which had a good therapeutic effect on osteoporosis. 

### Magnetic targeting

Li et al. [99] prepared NPs-containing exosomes by co-incubating MSCs with superparamagnetic iron oxide nanoparticles (SPIONs) for 16 h. Under the effect of the external magnetic field, the number of exosomes aggregated at the skin injury site was significantly increased and a better repair effect was achieved. Due to the small number of EVs produced by MSCs daily, it is difficult to collect sufficient quantities for use in clinical studies. Kim et al. [100, 101] prepared exosome-mimetic nanovesicles containing SPIONs by co-incubating MSCs with NPs and then extruding the cells using serial nano-porous membranes. Guided by an external magnetic field, they successfully produced significant targeted therapeutic effects on ischemic stroke and spinal cord injury. One safety concern that EVs can avoid compared to MSCs is the risk of vascular embolism caused by the accumulation of large amounts of MSCs in the local vascular lumen under the influence of external high-intensity magnetic fields.

## Ovary-specific targets and antibodies

After summarizing the ovarian targeting strategies for MSCs and MSCs-derived EVs, how to find ovarian-specific targets is particularly important. In my opinion, ovary-specific targets need to fulfill the following two characteristics: (1) the target must be a molecule on the surface of the cell membrane, such as a membrane receptor or ligand; (2) the target needs to be ovary-specific or highly expressed.

For example, anti-CD19 chimeric antigen receptor T (CAR-T) cell therapy for hematologic malignancies has undoubtedly achieved impressive results, and the key to its success lies in the selection of the specific target (CD19, expressed in more than 95% of B-cell malignant tumors) as well as in the continuous innovation of the CAR structure [102]. The molecular structure of CAR consists of four main parts: the extracellular domain, the hinge region, the transmembrane domain and the intracellular domain. The extracellular domain is the antigen-binding region, which contains the key molecule scFv to realize the targeting function. Notably, the level of affinity of scFv determines the tumor-targeting killing ability of CAR-T as well as off-target toxicity [103]. Therefore, the selection of specific targets and the production of high-affinity antibodies (including scFv and nanobody) will greatly affect the target repair ability of the ovary as well as the target delivery efficiency.

### The selection of ovarian-specific targets

Specifically, the selection of specific targets needs to focus on specific cell types of the ovary, such as oocytes, granulosa cells (GCs), and theca cells. Considering the presence of zona pellucida and reproductive safety concerns, targeting oocytes may need to be considered with caution. Granulosa cells, which are present at different follicular stages, adjacent to the oocyte and perform important functions, are a good choice. Following the gene expression data from the National Center for Biotechnology Information (NCBI), combined with published references related to GCs marker or single-cell sequencing data, screening and comparison of membrane proteins highly expressed in GCs and further validation at the protein level in conjunction with basic experiments would be one of the ideas to screen ovary-specific targets, such as FSHR, a common marker for GCs.

### The production of high-affinity antibodies

On the basis of a good selection of ovarian-specific targets, the production, optimization and validation of high-affinity antibodies will be involved. Current technologies for antibody development are relatively mature, such as humanization of monoclonal antibodies, phage display, single B-cell antibody technology and affinity maturation [104]. Despite the relative maturity of the technology, it is important to note the cost of the time required for the development of high-affinity antibodies and the risk of failure.

## Clinical trials

According to ClinicalTrials.gov, as of February 14, 2024, 21 clinical trials of MSCs and MSCs-derived EVs for the treatment of POI were registered. Of these trials, 1 trial is terminated, 6 trials are already completed, and recruitment is going on for 3 trials (Table 3). Notably, most of the interventions with MSCs were ovarian rather than intravenous injections. Therefore, considering the safety and simplicity of intravenous injection, the development of ovarian-targeted MSCs or MSCs-derived EVs is of great value for clinical applications.

**Table 3:** Clinical trials related MSCs and MSCs-derived EVs therapy performed or underway for POI

Trial ID	Enrollment	Study Status	Interventions	Phases	Start Date	Locations	Out come of Trial
NCT02151890	10	Completed	Laparoscopic stem cells transplantation	Phase1|Phase2	2012/3/1	Al Azhar University, Egypt	No results posted
NCT01742533	40	Unknown	hUMSCs, hCBMNCs transplantation combined HRT	Phase1|Phase2	2012/3/1	Shenzhen People's Hospital, China	No results posted
NCT02062931	60	Unknown	Laparoscopic autologous BMSCs transplantation	Phase1|Phase2	2012/3/1	Al Azhar University, Egypt	No results posted
NCT02372474	112	Completed	Laparoscopic autologous BMSCs transplantation	Phase1|Phase2	2012/3/1	Al Azhar University, Egypt	No results posted
NCT01853501	4	Unknown	Autologous ADSCs ovary injection	Phase4	2012/9/1	The affiliated Drum Towel Hospital, China	No results posted
NCT02043743	60	Unknown	Autologous BMSCs ovary injection	Phase1|Phase2	2014/1/1	El-Rayadh Fertility Center, Egypt	No results posted
NCT03069209	50	Unknown	Autologous BMSCs ovary injection	Phase1|Phase2	2015/1/1	Stem Cells Arabia, Jordan	No results posted
NCT02151890	10	Completed	Laparoscopic stem cells transplantation	Phase1|Phase2	2012/3/1	Al Azhar University, Egypt	No results posted
NCT02603744	9	Unknown	Autologous ADSCs ovary injection	Phase1|Phase2	2015/6/1	Royan Institute, Iran	No results posted
NCT02644447	23	Completed	hUMSCs with injectable collagen scaffold transplantation	Phase1|Phase2	2015/10/1	The affiliated Drum Towel Hospital, China	No results posted
NCT03033277	320	Unknown	Intraovarian injection of hUMSCs under the guidance of ultrasonic	Phase1|Phase2	2016/2/1	Institute of Zoology, Chinese Academy of Sciences, China	No results posted
NCT02696889	3	Completed	Laparoscopic autologous BMSCs ovary injection	NA	2016/2/6	University of Illinois at Chicago, United States	No results posted
NCT02779374	10	Terminated	Autologous BMSCs intravenous injection	NA	2016/7/1	South Valley University, Egypt	No results posted
NCT03816852	12	Suspended	hUMSCs intravenous injection	Phase2	2018/10/1	Henan Provincial People's Hospital, China	No results posted
NCT05138367	20	Completed	UCA-PSCs or WJ-MSCs ovary injection under the guidance of ultrasonic	Phase1	2018/12/1	The affiliated Drum Towel Hospital, China	No results posted
NCT03877471	28	Unknown	hESC-MSCs ovary injection under the guidance of ultrasonic	Phase1	2019/4/3	The First Affiliated Hospital of Zhengzhou University, China	No results posted
NCT05308342	66	Recruiting	hUMSCs ovary injection under the guidance of ultrasonic	NA	2019/11/20	The affiliated Drum Towel Hospital, China	No results posted
NCT05494723	6	Not yet recruiting	YB-1113 (hUMSCs) intravenous injection	Phase1	2024/1/9	Bright Cell, Inc	No results posted
NCT06132542	10	Not yet recruiting	ADSCs ovary injection under the guidance of ultrasonic	Phase1	2024/1/15	Mongolian National University of Medical Science, Mongolia	No results posted
NCT06202547	10	Recruiting	BMSCs derived EVs ovary injection under the guidance of ultrasonic	Phase1|Phase2	2023/2/20	Royan Institute, Iran	No results posted
NCT06072794	9	Recruiting	VL-PX10 (hPMSCs derived exosmes) intravenous injection	Phase1	2023/10/6	Optimal Health Associates, United States	No results posted

## Future challenges

### Homogeneity of MSCs and MSCs-derived EVs

Despite the large number of clinical trials related to MSCs, there are still some concerns and questions about the homogeneity of MSCs products. Studies have shown that the individual origin of MSCs (e.g., age, sex), tissue source, culture conditions, isolation methods, culture generations, and subpopulations of MSCs can lead to differences in gene expression profiles as well as therapeutic effects of MSCs [105–108]. The homogeneity as well as therapeutic effects of EVs can also be influenced by the characteristics of MSCs, purification methods, engineering approaches, and storage methods [109]. Therefore, the design of preclinical and clinical trials needs to take into account the possible impact of the above differences and ensure that MSCs and EVs products have strict production standards and quality testing procedures.

To ensure homogeneity and product quality, the following four programs are indispensable: (1) ensure that each product has clear and traceable biological information about the healthy donor; (2) establish strict operation standards and management procedures for MSCs/EVs collection, isolation, cultivation, cell line establishment, preservation and transportation, as well as personnel training, instrument use and environmental maintenance; (3) to ensure the homogeneity of the product batches, try to establish the cell bank production, and pay attention to the homogeneity of the donor material; (4) the quality research of the product should be comprehensive and continuous, including the analysis of cellular properties, physical and chemical properties, purity and impurity analysis, safety analysis and biological activity analysis.

### Yield of MSCs-derived EVs

As mentioned above, there may be differences in homogeneity and therapeutic effects of EVs derived from different culture generations of MSCs. As cells isolated and cultured from normal tissues, MSCs are limited in number as well as in the number of culture generations. For example, an ongoing clinical trial requires 5–20 μg of MSCs per participant, twice weekly for 12 weeks (ClinicalTrials.gov Identifier: NCT04388982), and the total number of exosomes required for 9 participants would take several weeks to produce. Therefore, the yield of EVs is largely limited by the quantity and quality of MSCs under conventional culture conditions, and there is a need to develop new methods to increase the yield of MSCs-derived EVs.

Conventional methods to enhance EVs secretion are difficult to meet the needs of large-scale EVs production, such as (1) genetic engineering based on exosome biogenesis and release pathways, (2) pretreatment of parental cells or addition of different additives to the culture medium, and (3) 3D cell culture based on biomaterials [110]. For large-scale production, the current mainstream means is to 3D culture large quantities of MSCs in rotating flasks based on microcarriers. Haraszti et al*.* showed that this method in combination with tangential flow filtration (TFF, a method of concentrating proteins from large quantities of media) can increase the yield by a factor of about 140 compared to the traditional 2D culture in combination with ultracentrifugation [111]. In addition, this method is easy to meet the Good Manufacturing Practice (GMP) requirements in production and has a very promising clinical application.

Notably, the extrusion of MSCs by serial nano-porous membranes allows simple, easy, and efficient access to large amounts of exosome-mimetic nanovesicles [112]. It is undeniable that there are differences between exosome-mimicking nanoparticles and real culture-obtained exosomes, but due to its great yield and ease of production, it has great application prospects in the field of targeted delivery.

### Purity of MSCs-derived EVs

Efficient isolation and purification of EVs and effective removal of contaminating proteins and other possible contaminants are necessary to reduce the heterogeneity of EVs. EVs isolation methods include ultracentrifugation, ultrafiltration, density gradient, size-exclusion chromatography, immunoaffinity capture, and commercial reagents [113, 114]. Although a large number of isolation and purification methods have been developed, no single method is perfect.

For example, the methods in order from highest to lowest yield are approximately commercial reagents, ultrafiltration and ultracentrifugation, yet the order of purity is almost reversed [114]. Ultracentrifugation, while obtaining high-purity exosomes for clinical use, has the disadvantages of high cost, cumbersome operation, damage of isolated vesicles and protein aggregation. Immunoaffinity capture and size-exclusion chromatography, while obtaining relatively high-purity EVs, are difficult to meet the standards required for clinical use and have low yield. Therefore, exosome purification methods limit the standardization and large-scale production of exosomes and represent a great challenge for the future clinical use of exosomes.

### Long-term safety and efficacy assessment

Although there have been a large number of preclinical studies of MSCs and MSCs-derived EVs for the treatment of POI in recent years (Tables 1, 2), most of these studies have emphasized the restoration of ovarian function and improvement of fertility, with little focus on long-term safety and efficacy. Especially for MSCs or MSCs-derived EVs that are genetically engineered to enhance ovarian homing/targeting, the potential safety issues (tumorigenicity as well as immunogenicity) should be more concerned. Especially in the case of oocytes, the risk of introducing exogenous genes is something that needs to be considered extremely carefully.

Therefore, for the treatment of POI based on MSCs or MSCs-derived EVs, it is necessary to conduct more safety experiments on the parents and offspring, such as the long-term changes in body weight, blood routine, blood biochemistry, liver and kidney functions, ovarian function, fertility, tumor formation and so on, as well as the growth and development ability, learning ability, reproduction ability, and even genome changes of the offspring of the experimental animals. The above research will be of great significance to support more clinical trials and future clinical translation.

## Conclusions

The inherent ability of MSCs to sites of injury to secrete therapeutic mediators (including EVs) makes them a bright star for POI therapy. Due to the genetically manipulable as well as cargo loading properties of MSCs and MSCs-derived EVs, precision targeted therapies offer new hopes and challenges for the treatment of POI.

The administration route of MSCs and MSCs-derived EVs determines the biodistribution, therapeutic efficacy, and possible safety concerns raised after injection. To improve the ovarian targeting ability of MSCs and MSCs-derived EVs, a series of engineering approaches were evaluated, such as upregulating chemokine receptor expression or conjugating targeting peptide/nanobody/scFv by means of genetic engineering, surface modification, and pretreatment. In addition, there is also hope for aggregation of MSCs and MSCs-derived EVs to the damaged ovaries by means of magnetic targeting, with the premise of controlling the location and intensity of the magnetic field. Despite so many encouraging approaches, several challenges remain for the future clinical application of MSCs and their derived EVs, such as the review of homogeneity, the difficulty of mass production of EVs, the challenge of purification of EVs and insufficient conduct of preclinical trials.

Therefore, more advanced approaches are still needed to expand the potential of MSCs and MSCs-derived EVs in POI-targeted therapy. In the future, we look forward to more ovarian-targeted research strategies to achieve high-quality controlled targeted damage repair as well as targeted cargo delivery, bringing new hope for the POI population.

## Data Availability

Not applicable.

## Abbreviations

mBMSCs: Mouse bone mesenchymal stem cells
hUMSCs: Human umbilical cord mesenchymal stem cells
hAMSCs: Human amniotic mesenchymal stem cells
hAFMSCs: Human amniotic fluid mesenchymal stem cells
hADSCs: Human adipose mesenchymal stem cells
hCBMNCs: Human cord blood mononuclear cells
UCA-PSCs: Perivascular stem cells derived from umbilical arteries
WJ-MSCs: Mesenchymal stem cells derived from Wharton's jelly
MenSCs: Menstrual-derived mesenchymal stem cells
hESC-MSCs: Human embryonic stem cell derived mesenchymal stem cells
hPMSCs: Human placenta mesenchymal stem cells
CTX: Cyclophosphamide
CDDP: Cisplatin
BUS: Busulfan
GCs: Granulosa cells
ROS: Reactive oxygen species

## References

[1] Nelson LM (2009). Clinical practice. Primary ovarian insufficiency. N Engl J Med.

[2] Ebrahimi M, Akbari Asbagh F (2011). Pathogenesis and causes of premature ovarian failure: an update. Int J Fertil Steril.

[3] Stuenkel CA, Gompel A (2023). Primary ovarian insufficiency. N Engl J Med.

[4] Chon SJ, Umair Z, Yoon MS (2021). Premature ovarian insufficiency: past, present, and future. Front Cell Dev Biol.

[5] Ghahremani-Nasab M, Ghanbari E, Jahanbani Y, Mehdizadeh A, Yousefi M (2020). Premature ovarian failure and tissue engineering. J Cell Physiol.

[6] Sullivan SD, Sarrel PM, Nelson LM (2016). Hormone replacement therapy in young women with primary ovarian insufficiency and early menopause. Fertil Steril.

[7] Chang CL (2023). Facilitation of ovarian response by mechanical force-latest insight on fertility improvement in women with poor ovarian response or primary ovarian insufficiency. Int J Mol Sci.

[8] Telfer EE, Andersen CY (2021). In vitro growth and maturation of primordial follicles and immature oocytes. Fertil Steril.

[9] Vo KCT, Kawamura K (2021). In vitro activation early follicles: from the basic science to the clinical perspectives. Int J Mol Sci.

[10] Esfandyari S, Chugh RM, Park HS, Hobeika E, Ulin M, Al-Hendy A (2020). Mesenchymal stem cells as a bio organ for treatment of female infertility. Cells.

[11] Hoang DM, Pham PT, Bach TQ, Ngo ATL, Nguyen QT, Phan TTK, Nguyen GH, Le PTT, Hoang VT, Forsyth NR (2022). Stem cell-based therapy for human diseases. Signal Transduct Target Ther.

[12] Kabat M, Bobkov I, Kumar S, Grumet M (2020). Trends in mesenchymal stem cell clinical trials 2004–2018: is efficacy optimal in a narrow dose range?. Stem Cells Transl Med.

[13] He Y, Chen D, Yang L, Hou Q, Ma H, Xu X (2018). The therapeutic potential of bone marrow mesenchymal stem cells in premature ovarian failure. Stem Cell Res Ther.

[14] Li Z, Zhang M, Tian Y, Li Q, Huang X (2021). Mesenchymal stem cells in premature ovarian insufficiency: mechanisms and prospects. Front Cell Dev Biol.

[15] González-González A, García-Sánchez D, Dotta M, Rodríguez-Rey JC, Pérez-Campo FM (2020). Mesenchymal stem cells secretome: the cornerstone of cell-free regenerative medicine. World J Stem Cells.

[16] O'Brien K, Breyne K, Ughetto S, Laurent LC, Breakefield XO (2020). RNA delivery by extracellular vesicles in mammalian cells and its applications. Nat Rev Mol Cell Biol.

[17] Jeppesen DK, Zhang Q, Franklin JL, Coffey RJ (2023). Extracellular vesicles and nanoparticles: emerging complexities. Trends Cell Biol.

[18] van Niel G, D'Angelo G, Raposo G (2018). Shedding light on the cell biology of extracellular vesicles. Nat Rev Mol Cell Biol.

[19] Geng Z, Guo H, Li Y, Liu Y, Zhao Y (2023). Stem cell-derived extracellular vesicles: a novel and potential remedy for primary ovarian insufficiency. Front Cell Dev Biol.

[20] Wiklander OP, Nordin JZ, O'Loughlin A, Gustafsson Y, Corso G, Mäger I, Vader P, Lee Y, Sork H, Seow Y (2015). Extracellular vesicle in vivo biodistribution is determined by cell source, route of administration and targeting. J Extracell Vesicles.

[21] Zhang S, Huang B, Su P, Chang Q, Li P, Song A, Zhao X, Yuan Z, Tan J (2021). Concentrated exosomes from menstrual blood-derived stromal cells improves ovarian activity in a rat model of premature ovarian insufficiency. Stem Cell Res Ther.

[22] Krueger TEG, Thorek DLJ, Denmeade SR, Isaacs JT, Brennen WN (2018). Concise review: mesenchymal stem cell-based drug delivery: the good, the bad, the ugly, and the promise. Stem Cells Transl Med.

[23] Dominici M, Le Blanc K, Mueller I, Slaper-Cortenbach I, Marini F, Krause D, Deans R, Keating A, Prockop D, Horwitz E (2006). Minimal criteria for defining multipotent mesenchymal stromal cells. The International society for cellular therapy position statement. Cytotherapy.

[24] Cui L, Bao H, Liu Z, Man X, Liu H, Hou Y, Luo Q, Wang S, Fu Q, Zhang H (2020). hUMSCs regulate the differentiation of ovarian stromal cells via TGF-β(1)/Smad3 signaling pathway to inhibit ovarian fibrosis to repair ovarian function in POI rats. Stem Cell Res Ther.

[25] Lu X, Bao H, Cui L, Zhu W, Zhang L, Xu Z, Man X, Chu Y, Fu Q, Zhang H (2020). hUMSC transplantation restores ovarian function in POI rats by inhibiting autophagy of theca-interstitial cells via the AMPK/mTOR signaling pathway. Stem Cell Res Ther.

[26] Cui L, Bao H, Zhu W, Tang Y, Luo Q, Si Y, Fu Q, Jiang Z (2022). hUMSCs transplantation regulates AMPK/NR4A1 signaling axis to inhibit ovarian fibrosis in POI rats. Stem Cell Rev Rep..

[27] Zhao Y, Ma J, Yi P, Wu J, Zhao F, Tu W, Liu W, Li T, Deng Y, Hao J (2020). Human umbilical cord mesenchymal stem cells restore the ovarian metabolome and rescue premature ovarian insufficiency in mice. Stem Cell Res Ther.

[28] Deng T, He J, Yao Q, Wu L, Xue L, Wu M, Wu D, Li C, Li Y (2021). Human umbilical cord mesenchymal stem cells improve ovarian function in chemotherapy-induced premature ovarian failure mice through inhibiting apoptosis and inflammation via a paracrine mechanism. Reprod Sci.

[29] Ling L, Feng X, Wei T, Wang Y, Wang Y, Wang Z, Tang D, Luo Y, Xiong Z (2019). Human amnion-derived mesenchymal stem cell (hAD-MSC) transplantation improves ovarian function in rats with premature ovarian insufficiency (POI) at least partly through a paracrine mechanism. Stem Cell Res Ther.

[30] Ling L, Hou J, Liu D, Tang D, Zhang Y, Zeng Q, Pan H, Fan L (2022). Important role of the SDF-1/CXCR4 axis in the homing of systemically transplanted human amnion-derived mesenchymal stem cells (hAD-MSCs) to ovaries in rats with chemotherapy-induced premature ovarian insufficiency (POI). Stem Cell Res Ther.

[31] Liu R, Zhang X, Fan Z, Wang Y, Yao G, Wan X, Liu Z, Yang B, Yu L (2019). Human amniotic mesenchymal stem cells improve the follicular microenvironment to recover ovarian function in premature ovarian failure mice. Stem Cell Res Ther.

[32] Fu X, He Y, Wang X, Peng D, Chen X, Li X, Wang Q (2017). Overexpression of miR-21 in stem cells improves ovarian structure and function in rats with chemotherapy-induced ovarian damage by targeting PDCD4 and PTEN to inhibit granulosa cell apoptosis. Stem Cell Res Ther.

[33] El-Derany MO, Said RS, El-Demerdash E (2021). Bone marrow-derived mesenchymal stem cells reverse radiotherapy-induced premature ovarian failure: emphasis on signal integration of TGF-β, Wnt/β-catenin and hippo pathways. Stem Cell Rev Rep.

[34] Wang Z, Wang Y, Yang T, Li J, Yang X (2017). Study of the reparative effects of menstrual-derived stem cells on premature ovarian failure in mice. Stem Cell Res Ther.

[35] Qu Q, Liu L, Cui Y, Liu H, Yi J, Bing W, Liu C, Jiang D, Bi Y (2022). miR-126-3p containing exosomes derived from human umbilical cord mesenchymal stem cells promote angiogenesis and attenuate ovarian granulosa cell apoptosis in a preclinical rat model of premature ovarian failure. Stem Cell Res Ther.

[36] Yang Z, Du X, Wang C, Zhang J, Liu C, Li Y, Jiang H (2019). Therapeutic effects of human umbilical cord mesenchymal stem cell-derived microvesicles on premature ovarian insufficiency in mice. Stem Cell Res Ther.

[37] Gao T, Chen Y, Hu M, Cao Y, Du Y (2023). MicroRNA-220-3p in human umbilical cord mesenchymal stem cell-secreted exosomes inhibits granulosa cell apoptosis by targeting KLF6 and ATF4-ATF3-CHOP pathway in POF mice. Apoptosis.

[38] Ding C, Zhu L, Shen H, Lu J, Zou Q, Huang C, Li H, Huang B (2020). Exosomal miRNA-17-5p derived from human umbilical cord mesenchymal stem cells improves ovarian function in premature ovarian insufficiency by regulating SIRT7. Stem Cells.

[39] Yang W, Zhang J, Xu B, He Y, Liu W, Li J, Zhang S, Lin X, Su D, Wu T, Li J (2020). HucMSC-derived exosomes mitigate the age-related retardation of fertility in female mice. Mol Ther.

[40] Gao T, Cao Y, Hu M, Du Y (2022). Human umbilical cord mesenchymal stem cell-derived extracellular vesicles carrying MicroRNA-29a improves ovarian function of mice with primary ovarian insufficiency by targeting HMG-Box transcription factor/Wnt/β-catenin signaling. Dis Markers.

[41] Huang B, Lu J, Ding C, Zou Q, Wang W, Li H (2018). Exosomes derived from human adipose mesenchymal stem cells improve ovary function of premature ovarian insufficiency by targeting SMAD. Stem Cell Res Ther.

[42] Sun B, Ma Y, Wang F, Hu L, Sun Y (2019). miR-644-5p carried by bone mesenchymal stem cell-derived exosomes targets regulation of p53 to inhibit ovarian granulosa cell apoptosis. Stem Cell Res Ther.

[43] Ding C, Qian C, Hou S, Lu J, Zou Q, Li H, Huang B (2020). Exosomal miRNA-320a is released from hAMSCs and regulates SIRT4 to prevent reactive oxygen species generation in POI. Mol Ther Nucleic Acids.

[44] Geng Z, Chen H, Zou G, Yuan L, Liu P, Li B, Zhang K, Jing F, Nie X, Liu T, Zhang B (2022). Human amniotic fluid mesenchymal stem cell-derived exosomes inhibit apoptosis in ovarian granulosa cell via miR-369-3p/YAF2/PDCD5/p53 pathway. Oxid Med Cell Longev.

[45] Cao RC, Lv Y, Lu G, Liu HB, Wang W, Tan C, Su XW, Xiong Z, Ma JL, Chan WY (2023). Extracellular vesicles from iPSC-MSCs alleviate chemotherapy-induced mouse ovarian damage via the ILK-PI3K/AKT pathway. Zool Res.

[46] Murphy MB, Moncivais K, Caplan AI (2013). Mesenchymal stem cells: environmentally responsive therapeutics for regenerative medicine. Exp Mol Med.

[47] Zhuang WZ, Lin YH, Su LJ, Wu MS, Jeng HY, Chang HC, Huang YH, Ling TY (2021). Mesenchymal stem/stromal cell-based therapy: mechanism, systemic safety and biodistribution for precision clinical applications. J Biomed Sci.

[48] Xunian Z, Kalluri R (2020). Biology and therapeutic potential of mesenchymal stem cell-derived exosomes. Cancer Sci.

[49] Ullah M, Liu DD, Thakor AS (2019). Mesenchymal stromal cell homing: mechanisms and strategies for improvement. iScience..

[50] Lee RH, Pulin AA, Seo MJ, Kota DJ, Ylostalo J, Larson BL, Semprun-Prieto L, Delafontaine P, Prockop DJ (2009). Intravenous hMSCs improve myocardial infarction in mice because cells embolized in lung are activated to secrete the anti-inflammatory protein TSG-6. Cell Stem Cell.

[51] Toma C, Wagner WR, Bowry S, Schwartz A, Villanueva F (2009). Fate of culture-expanded mesenchymal stem cells in the microvasculature: in vivo observations of cell kinetics. Circ Res.

[52] Fischer UM, Harting MT, Jimenez F, Monzon-Posadas WO, Xue H, Savitz SI, Laine GA, Cox CS (2009). Pulmonary passage is a major obstacle for intravenous stem cell delivery: the pulmonary first-pass effect. Stem Cells Dev.

[53] Jang S, Lee K, Ju JH (2021). Recent updates of diagnosis, pathophysiology, and treatment on osteoarthritis of the knee. Int J Mol Sci.

[54] Lai RC, Yeo RW, Tan KH, Lim SK (2013). Exosomes for drug delivery - a novel application for the mesenchymal stem cell. Biotechnol Adv.

[55] Poon W, Kingston BR, Ouyang B, Ngo W, Chan WCW (2020). A framework for designing delivery systems. Nat Nanotechnol.

[56] Fu P, Zhang J, Li H, Mak M, Xu W, Tao Z (2021). Extracellular vesicles as delivery systems at nano-/micro-scale. Adv Drug Deliv Rev.

[57] Gupta D, Liang X, Pavlova S, Wiklander OPB, Corso G, Zhao Y, Saher O, Bost J, Zickler AM, Piffko A (2020). Quantification of extracellular vesicles in vitro and in vivo using sensitive bioluminescence imaging. J Extracell Vesicles.

[58] Lázaro-Ibáñez E, Faruqu FN, Saleh AF, Silva AM, Tzu-Wen Wang J, Rak J, Al-Jamal KT, Dekker N (2021). Selection of fluorescent, bioluminescent, and radioactive tracers to accurately reflect extracellular vesicle biodistribution in vivo. ACS Nano.

[59] Aimaletdinov AM, Gomzikova MO (2022). Tracking of extracellular vesicles' biodistribution: new methods and approaches. Int J Mol Sci.

[60] Nowakowski A, Walczak P, Lukomska B, Janowski M (2016). Genetic engineering of mesenchymal stem cells to induce their migration and survival. Stem Cells Int.

[61] Wei W, Huang Y, Li D, Gou HF, Wang W (2018). Improved therapeutic potential of MSCs by genetic modification. Gene Ther.

[62] Wynn RF, Hart CA, Corradi-Perini C, O'Neill L, Evans CA, Wraith JE, Fairbairn LJ, Bellantuono I (2004). A small proportion of mesenchymal stem cells strongly expresses functionally active CXCR4 receptor capable of promoting migration to bone marrow. Blood.

[63] Safarzadeh Kozani P, Safarzadeh Kozani P, Rahbarizadeh F, Khoshtinat Nikkhoi S (2021). Strategies for dodging the obstacles in CAR T cell therapy. Front Oncol.

[64] Rossignoli F, Grisendi G, Spano C, Golinelli G, Recchia A, Rovesti G, Orsi G, Veronesi E, Horwitz EM, Dominici M (2019). Inducible Caspase9-mediated suicide gene for MSC-based cancer gene therapy. Cancer Gene Ther.

[65] Ramos CA, Asgari Z, Liu E, Yvon E, Heslop HE, Rooney CM, Brenner MK, Dotti G (2010). An inducible caspase 9 suicide gene to improve the safety of mesenchymal stromal cell therapies. Stem Cells.

[66] Marquez-Curtis LA, Gul-Uludag H, Xu P, Chen J, Janowska-Wieczorek A (2013). CXCR4 transfection of cord blood mesenchymal stromal cells with the use of cationic liposome enhances their migration toward stromal cell-derived factor-1. Cytotherapy.

[67] Hervás-Salcedo R, Fernández-García M, Hernando-Rodríguez M, Quintana-Bustamante O, Segovia JC, Alvarez-Silva M, García-Arranz M, Minguez P, Del Pozo V, de Alba MR (2021). Enhanced anti-inflammatory effects of mesenchymal stromal cells mediated by the transient ectopic expression of CXCR4 and IL10. Stem Cell Res Ther.

[68] Ling L, Hou J, Wang Y, Shu H, Huang Y (2022). Effects of low-intensity pulsed ultrasound on the migration and homing of human amnion-derived mesenchymal stem cells to ovaries in rats with premature ovarian insufficiency. Cell Transplant.

[69] Abdelrahman SA, Raafat N, Abdelaal GMM, Aal SMA (2023). Electric field-directed migration of mesenchymal stem cells enhances their therapeutic potential on cisplatin-induced acute nephrotoxicity in rats. Naunyn Schmiedebergs Arch Pharmacol.

[70] Sackstein R, Merzaban JS, Cain DW, Dagia NM, Spencer JA, Lin CP, Wohlgemuth R (2008). Ex vivo glycan engineering of CD44 programs human multipotent mesenchymal stromal cell trafficking to bone. Nat Med.

[71] Sulaiman SB, Chowdhury SR, Busra M, Abdul Rani RB, Mohamad Yahaya NHB, Tabata Y, Hiraoka Y, Haji Idrus RB, Hwei NM (2021). Type II collagen-conjugated mesenchymal stem cells micromass for articular tissue targeting. Biomedicines..

[72] Liao N, Zhang D, Wu M, Yang H, Liu X, Song J (2021). Enhancing therapeutic effects and in vivo tracking of adipose tissue-derived mesenchymal stem cells for liver injury using bioorthogonal click chemistry. Nanoscale.

[73] Chen X, Wang Q, Li X, Wang Q, Xie J, Fu X (2018). Heat shock pretreatment of mesenchymal stem cells for inhibiting the apoptosis of ovarian granulosa cells enhanced the repair effect on chemotherapy-induced premature ovarian failure. Stem Cell Res Ther.

[74] Wang Q, Li X, Wang Q, Xie J, Xie C, Fu X (2019). Heat shock pretreatment improves mesenchymal stem cell viability by heat shock proteins and autophagy to prevent cisplatin-induced granulosa cell apoptosis. Stem Cell Res Ther.

[75] Cheng J, Ruan X, Li Y, Du J, Jin F, Gu M, Zhou Q, Xu X, Yang Y, Wang H, Mueck AO (2022). Effects of hypoxia-preconditioned HucMSCs on neovascularization and follicle survival in frozen/thawed human ovarian cortex transplanted to immunodeficient mice. Stem Cell Res Ther.

[76] Liu H, Liu S, Li Y, Wang X, Xue W, Ge G, Luo X (2012). The role of SDF-1-CXCR4/CXCR7 axis in the therapeutic effects of hypoxia-preconditioned mesenchymal stem cells for renal ischemia/reperfusion injury. PLoS ONE.

[77] Ponte AL, Marais E, Gallay N, Langonné A, Delorme B, Hérault O, Charbord P, Domenech J (2007). The in vitro migration capacity of human bone marrow mesenchymal stem cells: comparison of chemokine and growth factor chemotactic activities. Stem Cells.

[78] Liu M, Yu W, Zhang F, Liu T, Li K, Lin M, Wang Y, Zhao G, Jiang J (2021). Fe(3)O(4)@polydopamine-labeled MSCs targeting the spinal cord to treat neuropathic pain under the guidance of a magnetic field. Int J Nanomedicine.

[79] Silva LHA, Silva MC, Vieira JB, Lima ECD, Silva RC, Weiss DJ, Morales MM, Cruz FF, Rocco PRM (2020). Magnetic targeting increases mesenchymal stromal cell retention in lungs and enhances beneficial effects on pulmonary damage in experimental silicosis. Stem Cells Transl Med.

[80] Li X, Wei Z, Wu L, Lv H, Zhang Y, Li J, Yao H, Zhang H, Yang B, Xu X, Jiang J (2020). Efficacy of Fe(3)O(4)@polydopamine nanoparticle-labeled human umbilical cord Wharton's jelly-derived mesenchymal stem cells in the treatment of streptozotocin-induced diabetes in rats. Biomater Sci.

[81] Hour FQ, Moghadam AJ, Shakeri-Zadeh A, Bakhtiyari M, Shabani R, Mehdizadeh M (2020). Magnetic targeted delivery of the SPIONs-labeled mesenchymal stem cells derived from human Wharton's jelly in Alzheimer's rat models. J Control Release.

[82] Huang Z, Shen Y, Sun A, Huang G, Zhu H, Huang B, Xu J, Song Y, Pei N, Ma J (2013). Magnetic targeting enhances retrograde cell retention in a rat model of myocardial infarction. Stem Cell Res Ther.

[83] Kobayashi T, Ochi M, Yanada S, Ishikawa M, Adachi N, Deie M, Arihiro K (2008). A novel cell delivery system using magnetically labeled mesenchymal stem cells and an external magnetic device for clinical cartilage repair. Arthroscopy.

[84] Yanai A, Häfeli UO, Metcalfe AL, Soema P, Addo L, Gregory-Evans CY, Po K, Shan X, Moritz OL, Gregory-Evans K (2012). Focused magnetic stem cell targeting to the retina using superparamagnetic iron oxide nanoparticles. Cell Transplant.

[85] Silva LH, Cruz FF, Morales MM, Weiss DJ, Rocco PR (2017). Magnetic targeting as a strategy to enhance therapeutic effects of mesenchymal stromal cells. Stem Cell Res Ther.

[86] Wang H, Alarcón CN, Liu B, Watson F, Searles S, Lee CK, Keys J, Pi W, Allen D, Lammerding J (2021). Genetically engineered and enucleated human mesenchymal stromal cells for the targeted delivery of therapeutics to diseased tissue. Nat Biomed Eng..

[87] Kamerkar S, LeBleu VS, Sugimoto H, Yang S, Ruivo CF, Melo SA, Lee JJ, Kalluri R (2017). Exosomes facilitate therapeutic targeting of oncogenic KRAS in pancreatic cancer. Nature.

[88] Wang S, Lei B, Zhang E, Gong P, Gu J, He L, Han L, Yuan Z (2022). Targeted therapy for inflammatory diseases with mesenchymal stem cells and their derived exosomes: from basic to clinics. Int J Nanomedicine.

[89] Ferreira D, Moreira JN, Rodrigues LR (2022). New advances in exosome-based targeted drug delivery systems. Crit Rev Oncol Hematol.

[90] Wu P, Zhang B, Ocansey DKW, Xu W, Qian H (2021). Extracellular vesicles: a bright star of nanomedicine. Biomaterials.

[91] Alvarez-Erviti L, Seow Y, Yin H, Betts C, Lakhal S, Wood MJ (2011). Delivery of siRNA to the mouse brain by systemic injection of targeted exosomes. Nat Biotechnol.

[92] Wang X, Chen Y, Zhao Z, Meng Q, Yu Y, Sun J, Yang Z, Chen Y, Li J, Ma T (2018). Engineered exosomes with ischemic myocardium-targeting peptide for targeted therapy in myocardial infarction. J Am Heart Assoc.

[93] Mentkowski KI, Lang JK (2019). Exosomes engineered to express a cardiomyocyte binding peptide demonstrate improved cardiac retention in vivo. Sci Rep.

[94] Tian Y, Li S, Song J, Ji T, Zhu M, Anderson GJ, Wei J, Nie G (2014). A doxorubicin delivery platform using engineered natural membrane vesicle exosomes for targeted tumor therapy. Biomaterials.

[95] Liang Y, Xu X, Li X, Xiong J, Li B, Duan L, Wang D, Xia J (2020). Chondrocyte-targeted MicroRNA delivery by engineered exosomes toward a cell-free osteoarthritis therapy. ACS Appl Mater Interfaces.

[96] Zou X, Yuan M, Zhang T, Wei H, Xu S, Jiang N, Zheng N, Wu Z (2019). Extracellular vesicles expressing a single-chain variable fragment of an HIV-1 specific antibody selectively target Env(+) tissues. Theranostics.

[97] Kooijmans SA, Aleza CG, Roffler SR, van Solinge WW, Vader P, Schiffelers RM (2016). Display of GPI-anchored anti-EGFR nanobodies on extracellular vesicles promotes tumour cell targeting. J Extracell Vesicles.

[98] Cui Y, Guo Y, Kong L, Shi J, Liu P, Li R, Geng Y, Gao W, Zhang Z, Fu D (2022). A bone-targeted engineered exosome platform delivering siRNA to treat osteoporosis. Bioact Mater.

[99] Li X, Wang Y, Shi L, Li B, Li J, Wei Z, Lv H, Wu L, Zhang H, Yang B (2020). Magnetic targeting enhances the cutaneous wound healing effects of human mesenchymal stem cell-derived iron oxide exosomes. J Nanobiotechnology.

[100] Kim HY, Kumar H, Jo MJ, Kim J, Yoon JK, Lee JR, Kang M, Choo YW, Song SY, Kwon SP (2018). Therapeutic efficacy-potentiated and diseased organ-targeting nanovesicles derived from mesenchymal stem cells for spinal cord injury treatment. Nano Lett.

[101] Kim HY, Kim TJ, Kang L, Kim YJ, Kang MK, Kim J, Ryu JH, Hyeon T, Yoon BW, Ko SB, Kim BS (2020). Mesenchymal stem cell-derived magnetic extracellular nanovesicles for targeting and treatment of ischemic stroke. Biomaterials.

[102] Huang R, Li X, He Y, Zhu W, Gao L, Liu Y, Gao L, Wen Q, Zhong JF, Zhang C, Zhang X (2020). Recent advances in CAR-T cell engineering. J Hematol Oncol.

[103] Duan Y, Chen R, Huang Y, Meng X, Chen J, Liao C, Tang Y, Zhou C, Gao X, Sun J (2021). Tuning the ignition of CAR: optimizing the affinity of scFv to improve CAR-T therapy. Cell Mol Life Sci.

[104] Lu RM, Hwang YC, Liu IJ, Lee CC, Tsai HZ, Li HJ, Wu HC (2020). Development of therapeutic antibodies for the treatment of diseases. J Biomed Sci.

[105] Hass R, Kasper C, Böhm S, Jacobs R (2011). Different populations and sources of human mesenchymal stem cells (MSC): a comparison of adult and neonatal tissue-derived MSC. Cell Commun Signal.

[106] Katsara O, Mahaira LG, Iliopoulou EG, Moustaki A, Antsaklis A, Loutradis D, Stefanidis K, Baxevanis CN, Papamichail M, Perez SA (2011). Effects of donor age, gender, and in vitro cellular aging on the phenotypic, functional, and molecular characteristics of mouse bone marrow-derived mesenchymal stem cells. Stem Cells Dev.

[107] Mushahary D, Spittler A, Kasper C, Weber V, Charwat V (2018). Isolation, cultivation, and characterization of human mesenchymal stem cells. Cytometry A.

[108] Chen P, Tang S, Li M, Wang D, Chen C, Qiu Y, Fang Z, Zhang H, Gao H, Weng H (2023). Single-cell and spatial transcriptomics decodes Wharton's jelly-derived mesenchymal stem cells heterogeneity and a subpopulation with wound repair signatures. Adv Sci (Weinh).

[109] Herrmann IK, Wood MJA, Fuhrmann G (2021). Extracellular vesicles as a next-generation drug delivery platform. Nat Nanotechnol.

[110] Qu Q, Fu B, Long Y, Liu ZY, Tian XH (2023). Current strategies for promoting the large-scale production of exosomes. Curr Neuropharmacol.

[111] Haraszti RA, Miller R, Stoppato M, Sere YY, Coles A, Didiot MC, Wollacott R, Sapp E, Dubuke ML, Li X (2018). Exosomes produced from 3D cultures of MSCs by tangential flow filtration show higher yield and improved activity. Mol Ther.

[112] Li YJ, Wu JY, Liu J, Xu W, Qiu X, Huang S, Hu XB, Xiang DX (2021). Artificial exosomes for translational nanomedicine. J Nanobiotechnol.

[113] Gandham S, Su X, Wood J, Nocera AL, Alli SC, Milane L, Zimmerman A, Amiji M, Ivanov AR (2020). Technologies and standardization in research on extracellular vesicles. Trends Biotechnol.

[114] Lai JJ, Chau ZL, Chen SY, Hill JJ, Korpany KV, Liang NW, Lin LH, Lin YH, Liu JK, Liu YC (2022). Exosome Processing and Characterization Approaches for Research and Technology Development. Adv Sci (Weinh).

